# Newborn Screen for X-Linked Adrenoleukodystrophy Using Flow Injection Tandem Mass Spectrometry in Negative Ion Mode

**DOI:** 10.3390/ijns8020027

**Published:** 2022-04-14

**Authors:** Tarek A. Teber, Brian J. Conti, Christopher A. Haynes, Amy Hietala, Mei W. Baker

**Affiliations:** 1 Newborn Screening Laboratory, Wisconsin State Laboratory of Hygiene, University of Wisconsin School of Medicine and Public Health, 465 Henry Mall, Madison, WI 53706, USA; tarek.teber@slh.wisc.edu (T.A.T.); brian.conti@slh.wisc.edu (B.J.C.); 2 Newborn Screening and Molecular Biology Branch, Centers for Disease Control and Prevention, 4770 Buford Hwy. NE, Atlanta, GA 30341, USA; cph7@cdc.gov; 3 Newborn Screening Laboratory, Minnesota Department of Health, St. Paul, MN 55164, USA; amy.hietala@state.mn.us; 4 Genetics and Metabolism Division, Department of Pediatrics, University of Wisconsin School of Medicine and Public Health, Madison, 1500 Highland Avenue, Madison, WI 53705, USA; 5 Center for Human Genomics and Precision Medicine, University of Wisconsin School of Medicine and Public Health, Madison, 1111 Highland Avenue, Madison, WI 53705, USA

**Keywords:** dried blood spots, flow injection analysis, C26:0-lysophosphatidylcholine, newborn screening, tandem mass spectrometry, X-linked adrenoleukodystrophy

## Abstract

X-linked adrenoleukodystrophy (X-ALD) is a genetic disorder caused by pathogenic variants in the ATP-binding cassette subfamily D member 1 gene (*ABCD1*) that encodes the adrenoleukodystrophy protein (ALDP). Defects in ALDP result in elevated cerotic acid, and lead to C26:0-lysophosphatidylcholine (C26:0-LPC) accumulation, which is the primary biomarker used in newborn screening (NBS) for X-ALD. C26:0-LPC levels were measured in dried blood spot (DBS) NBS specimens using a flow injection analysis (FIA) coupled with electrospray ionization (ESI) tandem mass spectrometry (MS/MS) performed in negative ion mode. The method was validated by assessing and confirming linearity, accuracy, and precision. We have also established C26:0-LPC cutoff values that identify newborns at risk for X-ALD. The mean concentration of C26:0-LPC in 5881 de-identified residual routine NBS specimens was 0.07 ± 0.02 µM (mean + 1 standard deviation (SD)). All tested true X-ALD positive and negative samples were correctly identified based on C26:0-LPC cutoff concentrations for borderline between 0.15 µM and 0.22 µM (mean + 4 SD) and presumptive screening positive at ≥0.23 µM (mean + 8 SD). The presented FIA method shortens analysis run-time to 1.7 min, while maintaining the previously established advantage of utilizing negative mode MS to eliminate isobaric interferences that could lead to screening false positives.

## 1. Introduction

X-linked adrenoleukodystrophy (X-ALD) is a genetic disorder with a birth prevalence of 1:14,700 and is caused by pathogenic variants in the *ABCD1* gene located at Xq28 [1,2]. Notably, there is no established genotype and phenotype correlation in X-ALD [2,3]. While a range of clinical manifestations are observed in both sexes, approximately 30% of males who possess a pathogenic *ABCD1* variant become afflicted with severe cerebral adrenoleukodystrophy (CALD) characterized by the demyelination of nerve cells within the brain [3,4,5,6,7,8]. Symptoms include dementia, seizures, loss of muscle control, hearing loss, and learning disabilities, but it ultimately leads to death within a few years. Standard medical care, autologous hematopoietic stem cell transplantation, and newer gene therapeutic stem cell strategies are highly effective, but only if patients are treated before permanent damage occurs to the brain [9,10,11,12]. Magnetic resonance imaging can be used to monitor for the onset of CALD before significant clinical symptoms occur, which signals the need for prompt treatment [13,14,15,16,17]. In recognition that early treatment is critical to positive patient outcome, X-ALD was added to the United States federal recommended uniform newborn screening panel (RUSP) in February 2016 [18].

C26:0-LPC has been used as a biomarker in NBS for X-ALD based on its high-levels of accumulation in patients possessing a pathogenic variant in *ABCD1* and the associated defect in ALDP activity [19,20,21,22]. MS/MS methods to measure C26:0-LPC levels include FIA in positive ion mode, though it has become apparent that this approach yields high levels of false positives [2,19,23,24,25,26]. For example, New York State’s newborn screening program initiated X-ALD screening in 2013 and first tier testing with FIA identified 6570 babies with potentially high levels of C26:0-LPC out of 365,000. A second tier test, which utilized a reverse phase liquid chromatography (LC) column, was necessary to separate out isobaric interferences to achieve the necessary specificity, which, for New York, reduced the number of potentially positive results to 33 [23]. A 3 min, negative mode LC-MS/MS method, introduced in 2012, eliminated detection of these interferences altogether, which has dramatically led to no false positive identifications when a borderline cutoff is employed that triggers repeat DBS collection and testing [27,28,29]. Additional LC method optimizations yielded two-minute sample analyses using positive mode LC-MS/MS that maintained low false positive rates similar to New York’s second tier testing [30,31]. Here we report a FIA–MS/MS X-ALD screen using newborn DBSs that is adapted from the negative mode LC-MS/MS assay. The updated method meets all standard validation criteria needed for integration into high-throughput newborn screening programs. Removing the LC component simplifies the analysis, results in a runtime of 1.7 min per sample, and retains the higher specificity of the negative ion mode analysis.

## 2. Materials and Methods

### 2.1. Materials

The internal standard (IS) 1-hexacosanoyl-d4-2-hydroxy-sn-glycero-3-phosphocholine (d4-C26:0-LPC) and the analyte C26:0-LPC were purchased from Cambridge Isotope Laboratories (Tewksbury, MA, USA). HPLC grade methanol and acetonitrile were purchased from VWR International LLC (Batavia, IL, USA). Ammonium acetate was from Sigma (Saint Louis MO, USA). The initial development of the assay utilized DBSs from the Newborn Screening Quality Assurance Program of the Centers for Disease Control and Prevention (CDC) (Atlanta, GA, USA) that are certified for homogeneity, accuracy, and stability [32,33,34]. The provided DBS materials were created by enriching 50% hematocrit whole blood with 6 levels of C26:0-LPC and other common newborn screening analytes and dispensed onto Grade 903 filter paper. For method validation, separate DBSs were created in a similar manner from a 1:1 mixture of saline-washed red bloods cells and human plasma (American Red Cross) that were enriched with 0.1, 0.25, 0.5, 1.0, or 2.0 µM C26:0-LPC.

### 2.2. Extraction of C26:0-LPC

For each sample, one 3.2 mm diameter DBS punch was shaken with 100 µL of working solution (0.4 µM d4-C26:0-LPC in methanol) for 30 min at 31 °C in a polypropylene 96-well plate. An amount of 50 µL of the extract was mixed with an equal volume of mobile phase (50:50 methanol/ acetonitrile with 5 mM ammonium acetate) in a new plate for LC-MS/MS analysis.

### 2.3. FIA-MS/MS

A total of 10 µL of each sample was injected using a Shimadzu Nexera liquid chromatography system for inline analysis with a Sciex API4500 triple quadrupole mass spectrometer in negative mode with a Turbo V Electrospray Ion Source (Sciex, Framingham, MA). The flow rate started at 0.1 mL/min for 0.1 min, was then lowered to 0.08 mL/min for 0.7 min, and was raised to 0.8 mL/min for the final 0.2 min. Compound, scan and source MS multiple reaction monitoring (MRM) parameters are detailed in Table 1 and Table 2. C26:0-LPC concentration in the blood was determined by the following equation, where [IS] = [d4-C26:0-LPC] = 0.4 µM and DF (dilution factor) = 31.25:

$$\left[C26:0\;LPC\right]=\left(\frac{C26:0\;LPC\;peak\;area}{IS\;peak\;area}\right)*\left[IS\right]*\left(DF\right)$$

Integrated peak areas were obtained with Sciex MultiQuant 2.1. software (Sciex, Framingham MA, USA). The average analysis time per sample was 1.7 min, which included both injection and MS data acquisition.

Following sample acquisition each day, a 3 h negative mode cleaning method was executed at 0.2 mL/min with a mobile phase of 0.1% formic acid in 50:50 methanol/Milli-Q water and with a source temperature of 700 °C [35].

### 2.4. Linearity, Accuracy, and Precision

Linearity was assessed by measuring quintuplicate samples at eleven C26:0-LPC levels: 0.0125, 0.025, 0.05, 0.075, 0.1, 0.2, 0.4, 0.8, 1.0, 1.5, and 2.0 µM. To generate the method evaluation samples, pooled extracts from 2.0 µM C26:0-LPC-spiked DBSs were diluted (*v*/*v*) into extracts from non-enriched DBSs. Accuracy and precision were determined by measuring C26:0-LPC concentrations in quintuplicate at five levels (0.1, 0.25, 0.5, 1.0, 2.0 µM C26:0-LPC) on five separate days.

### 2.5. Ion Suppression, Filter Paper Matrix, and Carryover

Ion suppression was evaluated by comparing internal standard areas in neat extraction solution to those in control and newborn samples in 61 separate plates run over the course of two months. For carryover assessment, the C26:0-LPC peak area in 2 µM enriched samples were compared to that of internal standards (lacking C26:0-LPC) injected immediately afterwards. Four additional internal standard sample injections were then performed before repeating the experiment for a total of five replicates. Background C26:0-LPC values were obtained from the last internal standard injection before starting a new replicate. Blank filter paper punches were analyzed to evaluate the presence of this matrix.

### 2.6. Population Distribution and Cutoff Establishment

C26:0-LPC levels in the newborn population were retrospectively assessed using 5881 de-identified sequential residual newborn DBS specimens received from 21 August 2020 to 21 September 2020 in Wisconsin. Determination of cutoff was established using a set of de-identified and blinded NBS residual specimens that contained both X-ALD screening positive and X-ALD screening negative cases from Minnesota, with X-ALD status established by *ABCD1* gene sequencing. Ethical approvals from the Wisconsin State Laboratory of Hygiene, Minnesota Department of Health, and CDC were obtained for the use of a total of 5893 de-identified residual NBS blood spots for the validation of this method and the determination of cutoff values.

## 3. Results

### 3.1. Linearity, Accuracy, and Precision

**Figure 1:**
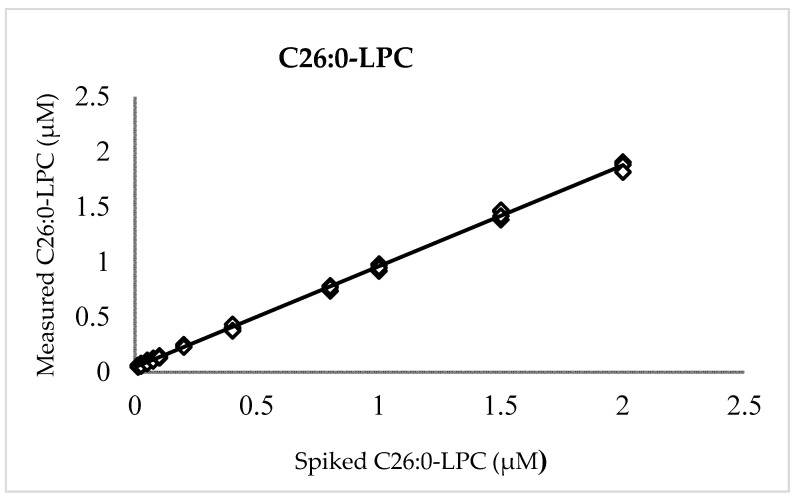
C26:0-LPC linearity. Measured vs. enriched concentrations of C26:0-LPC in quintuplicates at 0.0125, 0.025, 0.05, 0.075, 0.1, 0.2, 0.4, 0.8, 1.0, 1.5, and 2.0 µM.

A plot of measured versus enrichment C26:0-LPC quantities (Figure 1) exhibited linearity over the entire range of examined concentrations with a slope (recovery), intercept, and coefficient of determination (R²) of 0.916, 0.044, and 0.999, respectively. The positive intercept indicated a low level of background that can be attributed to either noise or, more likely, endogenous C26:0-LPC in the background matrix of blood cells and plasma observed in previous reports [2,9,11,12,20,25]. Unenriched DBSs, which were run separately, possessed C26:0-LPC values (*n* = 5) that averaged 0.039 µM ± 0.01 µM (SD), thereby verifying the presence of a background signal in unenriched DBS.

For accuracy and precision, 25 DBSs at each level of 0.1, 0.25, 0.5, 1.0, and 2.0 µM C26:0-LPC were measured over five days. The intra-run precision (CV) was no more than 9.6% for any level, while the inter-run precision fell to a maximum of 4.5%, indicating that measurement repeatability is acceptable for newborn screening and other clinical mass spectrometry assays [36,37]. The overall average C26:0-LPC values (±SD) were 0.113 ± 0.009 µM, 0.232 ± 0.011 µM, 0.463 ± 0.028 µM, 0.893 ± 0.048 µM, and 1.865 ± 0.092 µM for the levels denoted above, respectively. The 91.6% recovery and the background (0.044 µM) are not corrected in these calculations, because in newborn screening, presumptive positive newborns are distinguished from normal newborns by relative value differences, not absolute values, as described below. Regardless, the average deviation from the enriched amounts was no more than 13.2% for any level.

### 3.2. Ion Suppression, Filter Paper Matrix, and Carryover

In the five ion suppression experiments, the mean internal standard (IS) signal areas in the neat extraction solution, controls, and newborn specimens were 4.96 × 10^6^, 8.07 × 10^5^, and 8.06 × 10^5^ cps, respectively. The average ion suppression of the five replicates was 83.0% and 83.1% in controls and patient specimens with corresponding CVs of 4.5% and 4.4%. For carryover experiments, the C26:0-LPC signal in an internal standard injected immediately after 2 µM of C26:0-LPC-enriched samples was indistinguishable from the background. That is, the calculated average percent carryover was 0.0% with a standard deviation of 0.1%. Therefore, no carryover occurred. In mock samples, that is, blank filter paper spots lacking blood, no irrelevant signal was observed that added to the C26:0-LPC or IS intensities.

### 3.3. Population Distribution and Cutoff Establishment

**Figure 2:**
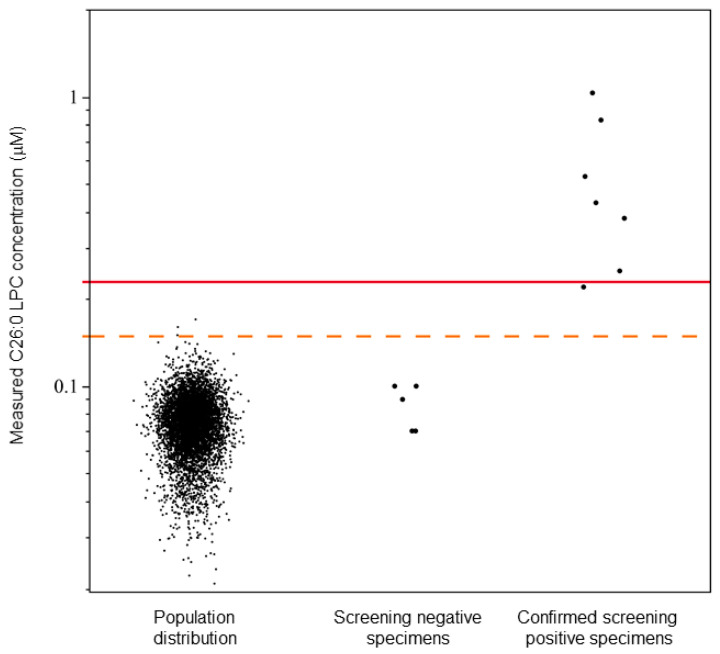
X-ALD newborn DBS study and cutoff establishment. Calculated C26:0-LPC levels are shown for a panel of Wisconsin newborns (*n* = 5881) and a panel of Minnesota newborn DBSs (*n* = 12) containing confirmed X-ALD cases (*n* = 7). Individual data points are shown. The dotted and solid lines indicate C26:0-LPC concentrations of 0.15 µM and 0.23 µM, respectively. C26:0-LPC concentrations are plotted on a log(10) scale.

In the set of 5881 de-identified sequential residual NBS specimens, a mean C26:0-LPC value of 0.07 µM was observed with a standard deviation of 0.02 µM (Figure 2). Based on four and eight standard deviations, C26:0-LPC concentrations between 0.15 and 0.22 µM, and ≥0.23 µM were determined to be borderline and presumptive positive X-ALD cutoff levels, respectively (Figure 2). Borderline samples would be subject to recollection of the DBSs for repeating testing, and presumptive positive X-ALD samples would be subject to confirmatory testing.

In the study comparing LC and FIA, the five true screening negative samples were correctly identified and possessed C26:0-LPC concentrations from 0.07 µM to 0.10 µM. Six true screening positive samples were correctly identified with C26:0-LPC concentrations from 0.22 µM to 0.83 µM, including one sample identified as borderline positive (Table 3).

## 4. Discussion

The negative ion mode MS/MS method validated here modifies a previously established LC method to a FIA method for X-ALD screening in newborns. Using the negative mode LC-MS/MS method described previously, the average X-ALD negative newborn has a C26:0-LPC DBS concentration of 0.09 µM, though positive mode MS/MS methods estimate the value anywhere from 0.033 to 0.6 depending on whether an LC column was used in the analysis [26,27,38,39]. Newborns with X-ALD, or alternatively other peroxisomal disorders that also manifest with elevated C26:0-LPC levels, have much higher average C26:0-LPC concentrations. In the most comparable negative mode LC-MS/MS study, the average C26:0-LPC concentration for X-ALD newborns was 1.13 µM, but notably the minimum was 0.32 µM, which was less than twice the level of the highest normal newborn (0.19 µM). Thus, it is critical to establish a method that can distinguish between X-ALD positive and negative newborns in such instances.

By converting the published LC-MS/MS negative C26:0-LPC method to an FIA format, significant ion suppression is introduced (~80–83%), presumably because a matrix is co-injected into the mass spectrometer. However, linearity, accuracy, and precision measurements still achieved similar values to those recommended by CLSI guidelines [36,37]. The linearity was established from 0.0125 to 2.0 µM C26:0-LPC, and this range sufficiently flanks both the screening borderline and positive cutoff values. A measurement of 0.039 µM was observed in standard prepared unenriched DBSs. Similar values were observed in blank (unenriched) DBSs prepared by the CDC (data not shown) and other X-ALD screening studies. The linearity study also indicated a C26:0-LPC recovery of 91.6%. Thus, at the 0.1 µΜ accuracy level near the population median, measured values deviated to 0.113 ± 0.009 µM due to a combination of background and recovery. Whereas at higher enrichment levels, the background was a smaller proportion of the absolute value, and thus C26:0-LPC measurements were accordingly lower than the enriched amounts. Though newborn screening does not rely upon absolute values to identify newborns at risk for X-ALD, the accuracy level only deviated from the true value by less than 15%, meeting standard validation requirements. The precision study demonstrated repeatable C26:0-LPC measurements of <10% CV at all levels examined (0.1, 0.25, 0.5, 1.0, and 2.0 µM), which arguably is more important than accuracy in a screening setting that relies upon relative measured values. Because the FIA method introduced a significant amount of extracted matrix material into the mass spectrometer, a cleaning method was executed on a daily basis in addition to routine system maintenance.

Descriptive statistics of C26:0-LPC values in a Wisconsin newborn sample population were used to establish cutoffs to identify newborns at risk for X-ALD. Using these cutoff values, such newborns were readily identified from a blinded panel of newborn DBSs whose disease status was revealed only after the cutoffs were determined. A single X-ALD patient in the blinded panel possessed borderline levels of C26:0-LPC, and three specimens (0.05%) were identified as borderline in the sampling of Wisconsin’s newborn population. Those three specimens would be subject to re-testing in duplicates in Wisconsin’s X-ALD screening program, with the possibility of screening negative or remaining as borderline. In other states, repeat collection and testing has been used to resolve whether these newborns truly have abnormal C26:0-LPC levels.

No specific interferences, as previously reported using positive ion mode flow injection [26,27], were observed when examining a basic mock sample (filter paper punch) that lacked blood. Our study of Wisconsin newborns (*n* = 5881) did not possess any positive specimens based on our established cutoff of 0.23 µM, which was not considered atypical given the X-ALD birth prevalence of 1 in 14,700 [2]. This also suggests unanticipated interferences are unlikely to exist, for example due to newborn bilirubin levels, total parent nutrition therapy, or abnormal hematocrit levels.

Although the findings in this report should be interpreted cautiously for other populations, the method presented here is expected to result in low levels of false positives, as observed in other states employing the LC-MS/MS negative mode X-ALD screening assay. In addition, flow injection analysis for C26:0-LPC will open up the possibility of multiplexing, though the adjustment of mobile phase pH and acetate ion concentrations will be required to integrate negatively- and positively-charged analytes into a single FIA program [39,40,41].

It is also worth mentioning that the expected C26:0-LPC values listed in Table 3 were acquired by a LC-MS/MS negative mode X-ALD screening assay. The closeness between the expected value and obtained value acquired by our FIA-MS/MS negative mode X-ALD screening assay further validates this new assay’s performance.

The previously established C26:0-LPC measurement in DBSs by negative mode LC-MS/MS methods has a satisfactory screening false positive rate, but it takes three minutes to process each specimen [27,28,29]. A positive mode LC-MS/MS method was reported with a runtime of two minutes per sample and features a low false positive rate [30,31]. The positive mode FIA-MS/MS method takes fewer than two minutes to process each specimen, but its false positive rate is of concern [2,23,24]. Here we report a new approach that measures C26:0-LPC in DBSs by negative mode FIA-MS/MS, which allows a low false positive rate and short sample processing time.

## Figures and Tables

**Table 1:** Compound dependent parameters for MS.

MRM	Q1 Mass (Da)	Q3 Mass (Da)	DP(V)	EP(V)	CE(V)	CXP(V)
C26:0-LPC	620.5	395.25	−189	−10	−45	−16
d4-C26:0-LPC	624.5	399.3	−189	−10	−45	−16

**Table 2:** Source dependent parameters for MS.

Curtain gas (psi)	28
Ion spray voltage (V)	−4500
Temperature (°C)	650
Gas1 (psi)	30
Gas2 (psi)	50
Collision assisted dissociation (psi)	7

**Table 3:** C26:0-LPC concentrations and associated outcome from comparison study.

Panel #	Measured C26:0-LPC (µM)		X-ALD Screening Outcome	
	Expected	Obtained	Expected	Obtained
1	1.06	1.03	Positive	Positive
2	0.06	0.07	Negative	Negative
3	0.07	0.09	Negative	Negative
4	0.26	0.25	Positive	Positive
5	0.18	0.22	Borderline	Borderline
6	0.10	0.10	Negative	Negative
7	0.10	0.10	Negative	Negative
8	0.06	0.07	Negative	Negative
9	0.44	0.43	Positive	Positive
10	0.74	0.83	Positive	Positive
11	0.49	0.53	Positive	Positive
12	0.33	0.38	Positive	Positive

## Data Availability

All study data have been reported in this manuscript.

## Disclaimer

The findings and conclusions in this report are those of the authors and do not necessarily represent the official position of the U.S. Department of Health and Human Services, or the U.S. Centers for Disease Control and Prevention (Division of Laboratory Sciences). The use of trade names and commercial sources is for identification only and does not constitute endorsement by the U.S. Department of Health and Human Services, or the U.S. Centers for Disease Control and Prevention (Division of Laboratory Sciences).
